# Identification and analysis of the connection network structure between the components of the immune system in children

**DOI:** 10.18699/vjgb-25-109

**Published:** 2025-12

**Authors:** D.S. Grebennikov, A.P. Toptygina, G.A. Bocharov

**Affiliations:** Marchuk Institute of Numerical Mathematics of the Russian Academy of Sciences (INM RAS), Moscow, Russia Moscow Center of Fundamental and Applied Mathematics at INM RAS, Moscow, Russia Sechenov First Moscow State Medical University of the Ministry of Health of the Russian Federation (Sechenov University), Moscow, Russia; Gabrichevsky Research Institute for Epidemiology and Microbiology, Moscow, Russia; Marchuk Institute of Numerical Mathematics of the Russian Academy of Sciences (INM RAS), Moscow, Russia Moscow Center of Fundamental and Applied Mathematics at INM RAS, Moscow, Russia Sechenov First Moscow State Medical University of the Ministry of Health of the Russian Federation (Sechenov University), Moscow, Russia

**Keywords:** immune system, immune status, correlation analysis, partial correlations, network topology, graphs, DSPC algorithm, иммунная система, иммунный статус, корреляционный анализ, частные корреляции, сетевая топология, графы, алгоритм DSPC

## Abstract

Identification of the connections between the various functional components of the immune system is a crucial task in modern immunology. It is key to implementing the systems biology approach to understand the mechanisms of dynamic changes and outcomes of infectious and oncological diseases. The data characterizing an individual’s immune status typically have a high-dimensional state space and a small sample size. To study the network topology of the immune system, we utilized previously published original data from Toptygina et al. (2023), which included measurements of the immune status in 19 healthy individuals (children, 9 boys and 10 girls, aged 1 to 2 years), i. e., the immune cells (42 subpopulations) obtained by flow cytometry; cytokine levels (13 types) obtained by multiplex analysis; and antibody levels (4 types) determined by using enzyme immunoassay. To correctly identify statistically significant correlations between the measured variables and construct the respective network graph, it is necessary to use an approach that takes into account the small size of the dataset. In this study, we implemented and analyzed an approach based on the regularized debiased sparse partial correlation (DSPC) algorithm to evaluate sparse partial correlations and identify the network structure of relationships in the immune system of healthy individuals (children) based on immune status data, which includes a set of indicators for subpopulations of immune cells, cytokine levels, and antibodies. For different levels of statistical significance, heatmaps of the partial correlations were constructed. The graph visualization of the DSPC networks was performed, and their topological characteristics were analyzed. It is found that with a limited measurements sample, the choice of a statistical significance threshold critically affects the structure of the partial correlations matrix. The final verification of the immunologically correct structure of the correlation-based network requires both an increase in the sample size and consideration of a priori mechanistic views and models of the functioning of the immune system components. The results of this analysis can be used to select the therapy targets and design combination therapies.

## Abstract (ru)

Идентификация связей между различными функциональными компонентами иммунной системы представляет собой чрезвычайно актуальную задачу современной иммунологии. Это необходимо для понимания механизмов динамики и исхода инфекционных и онкологических заболеваний при реализации системно-биологического подхода. Параметры, характеризующие иммунный статус человека, отличаются большой размерностью пространства состояний при малой мощности выборки. Для изучения сетевой топологии иммунной системы нами использованы ранее опубликованные оригинальные данные (Toptygina et al, 2023) измерений показателей иммунного статуса у 19 здоровых индивидуумов – детей, 9 мальчиков и 10 девочек, в возрасте от одного до двух лет: популяций иммунных клеток (42 субпопуляции), полученных с помощью проточной цитометрии; уровней цитокинов (13 типов), полученных методами мультиплексного анализа; уровня антител (4 типа), определенных с помощью иммуноферментного анализа. Для корректного (статистически значимого) определения корреляционных связей между измеряемыми переменными и построения графа сетевой топологии может быть использован подход, который учитывает малый размер множества данных. В нашей работе был реализован и исследован подход, в основе которого лежит регуляризированный алгоритм скорректированных разреженных частных корреляций (DSPC) оценивания разреженных частных корреляций и идентификации сетевой структуры взаимосвязей в иммунной системе по данным иммунного статуса здоровых детей, включающего набор показателей субпопуляций клеток иммунной системы, уровня цитокинов и антител. Для разных уровней статистической значимости были построены тепловые карты частных корреляций, выполнена визуализация сетей частных корреляций в виде графов и проведен анализ их топологических характеристик. Получено, что при ограниченной выборке измерений выбор порога для уровня статистической значимости имеет принципиальное значение для формирования матрицы частных корреляций. Окончательная верификация иммунологически корректной структуры связей требует как увеличения размера выборки, так и сопряжения с априорными механизменными представлениями и моделями функционирования компонент иммунной системы. Результаты могут быть использованы для выбора мишеней терапии и формирования комбинированных воздействий.

## Introduction

The human immune system functions to maintain the antigenic homeostasis of the body’s internal environment. It is a system with distributed parameters reflecting the spatial organization, phenotypic and clonal structure of its constituent cell populations. The cells of the immune system continuously interact with each other, and the balance of processes increasing or decreasing their activity underlies the development of productive or abortive reactions (Ng et al., 2013). Implementation of a systems biology approach to the investigation of the mechanisms determining the dynamics and outcome of infectious and oncological diseases requires identification of the structure of cellular interconnection networks in the immune system. An example of studying the connections network (network topology) between populations of cellular components of the immune system is provided in (Rieckmann et al., 2017), where the quantitative proteomics data were used for identification of the social architecture of immune cell interactions. The description of the network topology is associated with construction of a graph, with the vertices corresponding to specific cell populations of the immune system, and the edges representing connections of a diverse nature between the corresponding vertices.

To date, a large number (about 100 documented) of methods have been developed for analyzing the structural organization of intercellular interactions based on data of a diverse nature, including spatial and cellular transcriptomics, expression of ligand receptors, as well as intracellular signalling components (Armingol et al., 2024). They are used for the assessment of the connectivity indices or communication structures between cells, which provide the basis for building the graphs of connectivity networks. Both the biophysical and biochemical principles, and statistical data analysis methods in combination with machine learning, can be used to assess the strength of the intercellular connections.

The construction of a quantitative interactome of immune cells based on receptor proteins expressed on their surface is presented in (Shilts et al., 2022). It implements a number of graphs based on a set of physical connections between cells of the immune system in major human organs identified using multiplex immune and transcriptomic analysis technologies, genetic databases and biochemical methods for screening interactions between cells. Visualization of the transcriptome analysis data as a graph reflecting the genes co-expression is an integrative part of modern systemic vaccinology studies (Cortese et al., 2025).

The aim of our study was to implement a new approach to identifying the network structure of relationships in the immune system of a healthy individual based on the results of a correlation analysis of previously published data on the immune status of children aged one to two years. The data set includes the measurements of the immune status parameters, i. e. the subpopulations of immune cells, cytokine concentrations and antibody levels (Toptygina et al., 2023). The research objectives include the correlation analysis of children’s immune status data to build heatmaps of partial correlations, visualization of the partial correlations networks as graphs, and analysis of the topological characteristics of the graph models.

The present work consists of four sections. The “Materials and methods” section describes the specific features of the source data, methods of correlation analysis, the correlationbased approach to identifying a network structure of relationships between the immune status parameters, and examines the topological properties of the corresponding graphs. Principal components analysis is performed. The “Results” section presents the results of network construction for various threshold levels of statistical significance of the correlations, an immunological interpretation of the corresponding network topologies, and a robustness analysis. The results of the work are discussed in the “Discussion” section.

## Materials and methods

**Fig. 1.**
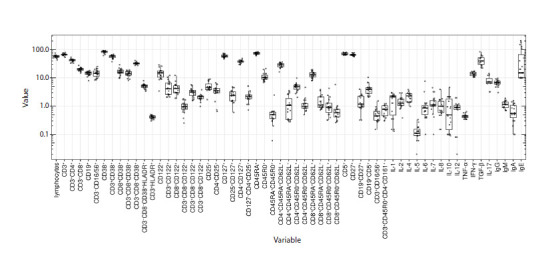
Data on immune status in healthy individuals – children aged one to two years (adapted from Toptygina et al., 2023). Individual measurements, median sample values, and 25–75 % quartiles are presented. The abscissa shows the names of the immune status indicators. The ordinate shows the percentage of cells (%), the levels of cytokines (pg/ml) and immunoglobulins A, M, G (g/l), IgE (IU/ml).

**Immune status data.** To study the network topology of the immune system, we used previously published original data (Toptygina et al., 2023). The data are a set of measurements of immune status indicators in 19 healthy individuals, i. e., children aged one to two years: populations of immune cells (42 subpopulations) obtained by flow cytometry; cytokine levels (13 types) obtained by multiplex analysis; antibody levels (4 types) determined by enzyme immunoassay. The data samples are summarized in Figure 1 as individual measurements, median values, and 25 and 75 % quartiles. The distribution of the indicators does not follow either the normal or the log-normal behavior.

The data on the immune status of children are characterized by a large dimensionality of the state space (59) and a small sample size (19 patients), which is typical for systems biology studies (Basu et al., 2017). If the sample size is large enough, one can use the approach based on partial correlations in order to determine the relationships between the immune status parameters. Otherwise, an approach that takes into account the small size of the data set has to be implemented to correctly determine statistically significantly correlations between the measured variables and construct a network topology graph. It should be noted that all the children belonged to the same age group from one to two years old, which in medical practice is not customary to subdivide further. Due to the small size of the group (19 people), additional division by gender (10 girls and 9 boys) would have reduced the statistical power below the critical level required for the method used in our study.

**Fig. 2.**
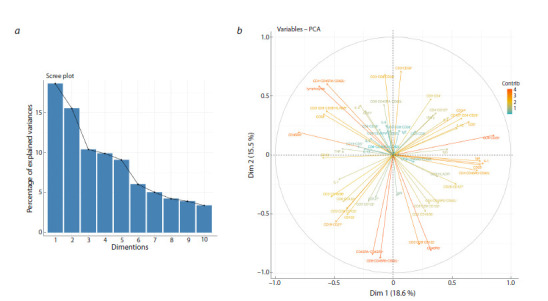
Principal component analysis: a – fraction of explained variance; b – composition of the first two principal components.

**Principal component analyses.** The principal component analysis (PCA) was performed using the prcomp function in the R language, the factoextra R package (version 1.0.7) was used for visualization. To perform the PCA, the data were standardized, and the variables TGF-β, IL-17, and CD3⁺CD45R0⁺CD4⁺CD161⁺ were excluded from the analysis due to missing data. The analysis of the principal components (PCs) did not reveal the possibility of explaining the variance of the data by a small number of the components (Fig. 2a), and no correlation-based clusters of immune status variables exist in the first two PCs (Fig. 2b).

**Methods of partial correlation analyses and reconstruction of the connection network. ** An alternative to the standard method of estimating partial correlations is an approach using regularization methods to estimate the matrix of partial correlations (Epskamp, Fried, 2018). The principle of regularization is based on the assumption that the number of connections in the constructed model network is significantly less than the number of observed variables, i. e. the real network is sparse. Accordingly, the LASSO method (Epskamp, Fried, 2018) is used as a regularizing correction that allows zeroing out insignificant correlations between variables (the number of edges in the graph). To analyze our data, we used this approach for the estimation of debiased sparse partial correlations matrix implemented in algorithm DSPC (Basu et al., 2017), which provides additional correction of estimates of the elements of the inverse covariance matrix, i. e. the elements of the partial correlations matrix. The estimates of the correlation matrix elements were represented as heatmaps and visualized as weighted networks, where the vertices (nodes) represent the immune status variables and the edges show correlations between them. The results of estimating the correlation-based relationships depend significantly on the algorithm parameters: 1) the value of the parameter λ for the regularization term in the form of ℓ1 norm of the inverse covariance matrix; 2) the choice of the statistical significance level p for the predicted correlation relationship. Below, we study the effect of the p-value on the network topology of connections in the immune system.

To calculate the sparse partial correlations using the DSPC method, we used the Java application CorrelationCalculator (version 1.0.1) developed in (Basu et al., 2017). The original data were normalized, i. e. logarithmically transformed and standardized. A graphical representation of statistically significant correlations (for p < 0.01; 0.05; 0.1; 0.15) in the form of heatmaps and graphs of correlation networks was performed using the R packages igraph (version 1.6.0) and ggplot2 (version 3.5.2). The topological characteristics of the correlation networks graphs were calculated using the igraph package in R (version 1.6.0).

## Results

In what follows, we study the effect of the p-value on the network topology of connections in the immune system. The conventionally considered statistical significance levels 0.01, 0.05, 0.1, 0.15 are analyzed.

**Heatmap and connection graph for p = 0.01**

**Fig. 3.**
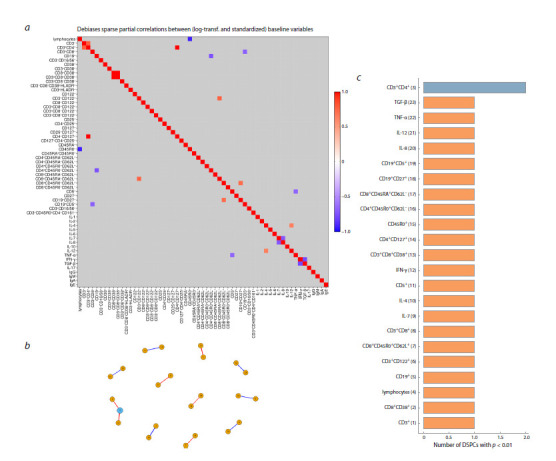
Heatmap and network graph of immunological parameters in healthy children at a statistical significance level of p = 0.01: a – heatmap of correlations between immune status indicators; b – graph of connections network at p = 0.01; c – characteristics of the complexity of the network of connections. Here and in Figures 4–6: the node numbers correspond to the immune status parameters shown in c. The ordinate names the immune status indicators. The abscissa shows the degrees of the graph nodes. Positive correlations (red lines), negative correlations (blue lines), the thickness of the edges is proportional to the absolute values of the DSPC coefficients. The color of the nodes corresponds to the node index, i. e. the number of significant correlations.

The heatmap of partial correlations between immune status parameters for healthy children at a statistical significance threshold p = 0.01 is presented in Figure 3a. The corresponding graph of the network is shown in Figure 3b. This graph has 23 nodes and 12 edges (connections). In fact, connectivity in the network is missing. Figure 3c shows the distribution of immune response indicators with respect to the number of identified links between them. The node with the maximum number (2 in total) of correlations represents the CD4 T cell population (CD3⁺CD4⁺).

**Heatmap and connection graph for p = 0.05**

**Fig. 4.**
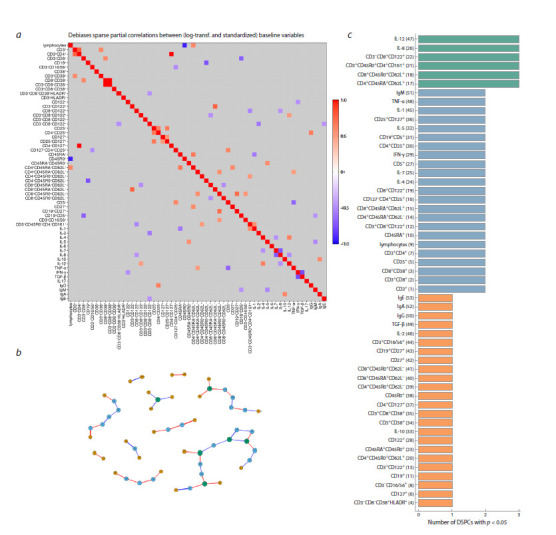
Heatmap and network graph of immunological parameters in healthy children at a statistical significance level of p = 0.05: a – heatmap of correlations between immune status indicators; b – graph of connections network at p = 0.05; c – characteristics of the complexity of the network of connections

The heatmap of correlations between immune status parameters for healthy children at a statistical significance threshold p = 0.05 is presented in Figure 4a. The corresponding network graph is shown in Figure 4b. This graph has 53 nodes and 44 edges (connections). The cohesion of individual network components is strengthened, but overall, it is absent. Figure 4c shows the distribution of immune response indicators with respect to the number of identified links between them. The nodes with the maximum number of correlations (called hubs) represent the proinflammatory cytokines IL-8, IL-12, and central memory T cells (CD4⁺CD45RA⁺CD62L⁺, CD8⁺CD45R0⁺CD62L⁺), Th17 (CD3⁺CD45R0⁺CD4⁺CD161⁺) and activated NK cells (CD3⁻CD8⁺CD122⁺). The maximum number of connections increases to three.

**Heatmap and connection graph for p = 0.1**

**Fig. 5.**
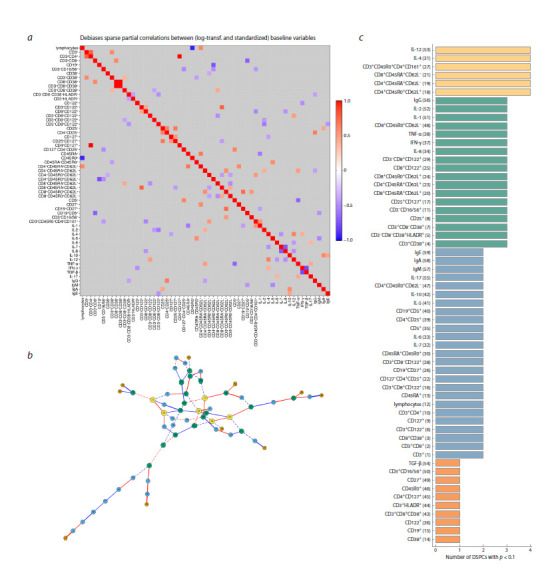
Heatmap and network graph of immunological parameters in healthy children at a statistical significance level of p = 0.1: a – heatmap of correlations between immune status indicators; b – graph of connections network at p = 0.1; c – characteristics of the complexity of the network of connections. Solid lines of the edges correspond to correlations with a significance level of p < 0.05, dashed lines, to p < 0.1.

The heatmap of correlations between immune status parameters for healthy children at a statistical significance threshold p = 0.1 is presented in Figure 5a. The corresponding network graph is shown in Figure 5b. This graph has 59 nodes and 69 edges (connections). Figure 5c shows the distribution of immune response indicators with respect to the number of identified links between them. The nodes with the maximum number of correlations (four in this case) represent the cytokines IL-4, IL-12 inducing the cellular and humoral immunity, the terminally differentiated effector memory T cells (CD4⁺CD45RA⁺CD62L⁻, CD8⁺CD45RA⁺CD62L⁻), and Th17 cells (CD3⁺CD45R0⁺CD4⁺CD161⁺).

**Heatmap and connection graph for p = 0.15**

**Fig. 6.**
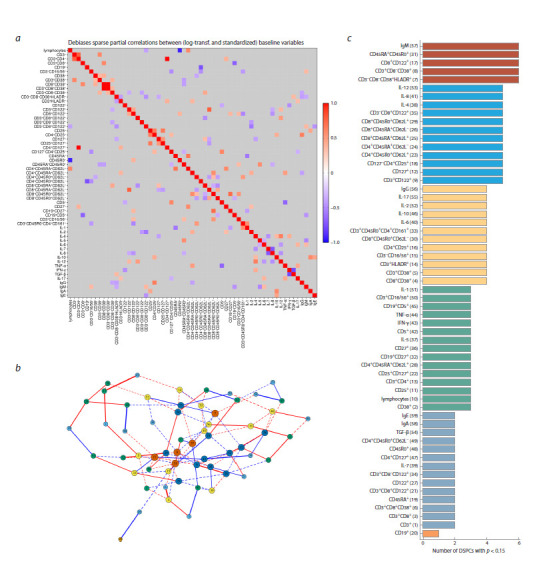
Heatmap and network graph of immunological parameters in healthy children at a statistical significance level of p = 0.15: a – heatmap of correlations between immune status indicators; b – graph of connections network at p = 0.15; c – characteristics of the complexity of the network of connections. Solid lines of the edges correspond to correlations with a significance level of p < 0.05, dashed lines, to p <0.15.

The heatmap of correlations between immune status parameters for healthy children at a statistical significance threshold p = 0.15 is presented in Figure 6a. The corresponding network graph is shown in Figure 6b. This graph has 59 nodes and 106 edges (connections). Figure 6c shows the distribution of immune response indicators with respect to the number of identified links between them. The nodes with the maximum number of correlations (hubs) represent the immunoglobulins IgM, plasma cells (CD3⁻CD8⁻CD38⁺HLADR⁺), activated T cells (CD3⁺CD8⁻CD38⁺, CD8⁺CD122⁺), and the doublepositive activated cells (CD45RA⁺CD45R0⁺) reflecting the transition from naive to memory cells. The maximum number of connections increases to six.

**Analysis of the robustness of correlation estimates**

**Fig. 7.**
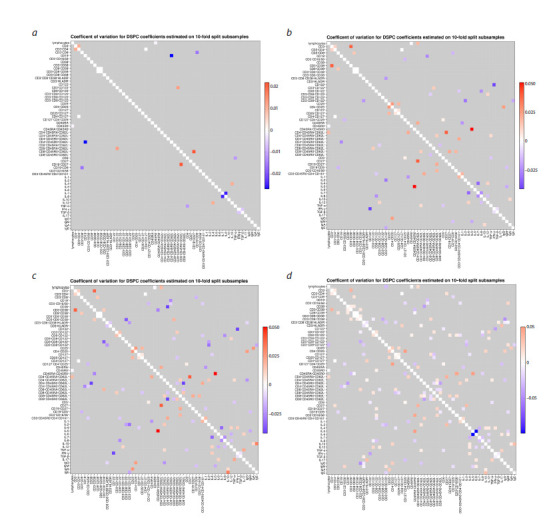
Matrices of estimates of the variation coefficients for four significance levels: p < 0.01 (a); p <0.05 (b); p < 0.1 (c); p < 0.15 (d).

To assess the stability of the obtained DSPC correlation coefficients in relation to the sample size, a procedure was performed for generating ten different subsamples according to the vfold10 scheme. In most cases, it corresponds to the selection of 17 out of 19 measurements. The coefficient of variation (the ratio of the standard deviation to the mean value) of the DSPC coefficients estimated from the generated subsamples was chosen as a measure of stability (robustness). The estimated coefficients of variation are shown in Figure 7 for four levels of statistical significance in the form of heatmaps. Importantly, their absolute values do not exceed 0.1.

**Comparative analysis of topological properties of graphs of correlations between indicators of immune status**

The Table shows the results of calculating the topological characteristics of the constructed graphs of correlation networks between immune status indicators for various thresholds of statistical significance. The following basic characteristics were considered: graph diameter, graph radius, girth of graph (the length of the smallest cycle contained in the graph), average path length, graph energy, spectral radius, edge density, clustering coefficient, average graph diversity (determined through entropy calculated by the weights of incident edges – the absolute values of the correlation coefficients DSPC), the number of separators, and the number of unconnected subgraphs.

**Table 1.**
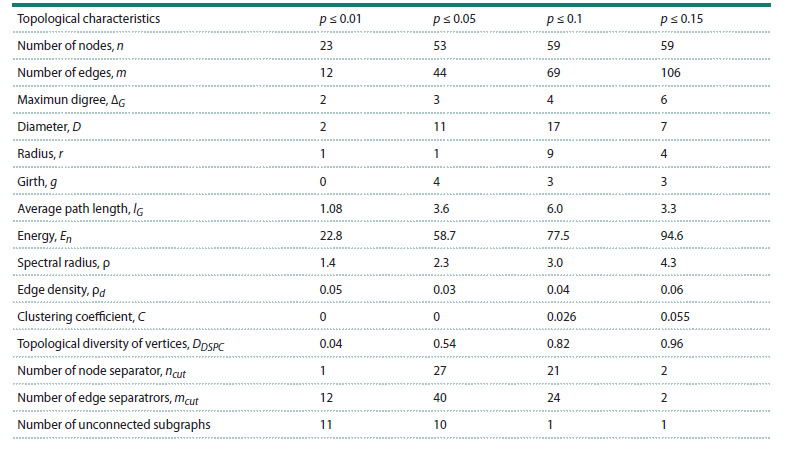
Comparative analysis of topological properties of graphs of correlations between indicators of immune status for various significance thresholds

The number of nodes, edges, and maximum node degrees grows with increasing statistical significance threshold. However, the graph diameter, radius, girth and average path length exhibit a non-monotonic dependence, initially increasing and then decreasing, which indicates a transformation of properties towards the “small world network” family. The graph energy and spectral radius increase monotonically with increasing threshold p. The clustering coefficient also increases, indicating that the graph nodes tend to cluster together. Interestingly, the number of cutting nodes and edges decreases at p = 0.15, which may indicate an increase in the robustness of the connections graph. As expected, the number of disconnected subgraphs decreases

## Discussion

Identification of the connection structures between the various functional components of the immune system is an extremely urgent task of modern immunology. This is due to an extremely high number of measured characteristics, with a relatively small sample size, reflecting the situation in big data biomathematics, called the “curse of dimensionality”. To analyze the relationships between immune status parameters, we implemented and analyzed an approach based on a regularized method for estimating sparse partial correlations implemented in the DSPC algorithm (Basu et al., 2017), which minimizes the number of false correlations. It is noted that the results of applying the algorithm may depend on the sample size, imputation of missing data, the nature of the true network structure and other aspects. Our work demonstrates that, given a limited sample size of measurements, an a priori assignment of the level of statistical significance is of fundamental importance for the formation of a matrix of partial correlations. Increasing the statistical significance threshold increases the complexity of the network topology generated by the DSPCbased approach. Final verification of the immunologically correct structure of connections requires both an increase in the sample size and conjugation with a priori mechanistic views and models of the functioning of the immune system components, i. e. the participation of clinical immunologists (Qiao et al., 2025). An important step in this direction was the development of the ImmunoGlobe tool for constructing and analyzing the network of interactions in the immune system (Atallah et al., 2020) using phenomenological information from the fundamental textbook “Janeway’s Immunobiology” (Murphy, Weaver, 2017).

The aim of this work is to implement and introduce a new method for identifying relationships between cellular and humoral components of the immune systems. Identification of the network relationships between elements of immune status is central to the systems immunology approach, but the relevant analytical tools remain undeveloped. All currently existing verified concepts of immune networks are limited to schemes with no more than three or four components (antigen presentation, differentiation pathways, paracrine and autocrine interactions). For this reason, it is not possible to uniquely select and verify one of the presented networks. If we adhere to the generally accepted level of significance (p = 0.05), then we should give preference to the network constructed in the section “Heatmap and graph of connections for p = 0.05”. Identifying the network structure of relationships between components of cellular and humoral immunity is a necessary element for the transition from a static description of immune status to a systems dynamics consideration of the maintenance of immune homeostasis

## Conclusion

The development of combination therapies for chronic diseases associated with induction of several components of the immune system requires understanding of the topology and strength of the structural connections in the system. Our study demonstrates for the first time that DSPC-based methods can be used to obtain consistent estimates of significant partial correlations for similar problems in a typical situation with a large number of measured immune status parameters and a small number of patients. Translation of the results into biomedical practice to address the challenges of personalized treatment and prevention of immune-dependent pathological processes requires an active participation of clinicians in order to determine therapy targets and quantitatively predict its effectiveness

## Conflict of interest

The authors declare no conflict of interest.
